# Prognostic factors for recurrent instability in recreational athletes following arthroscopic Bankart repair: a retrospective study with an average 4.1-year follow-up

**DOI:** 10.1186/s13102-024-00925-2

**Published:** 2024-06-24

**Authors:** Chunsen Zhang, Songyun Yang, Jiapeng Wang, Wenze Shao, Yizhou Huang, Xin Tang

**Affiliations:** 1 Sports Medicine Center, West China Hospital, Sichuan University, Chengdu, 610041 China; 2 Department of Orthopedics and Orthopedic Research Institute, West China Hospital, Sichuan University, Chengdu, 610041 China; 3 Department of Orthopedic Surgery and Orthopedic Research Institute, Laboratory of Stem Cell and Tissue Engineering, State Key Laboratory of Biotherapy, West China hospital, Sichuan University, Chengdu, 610041 China; 4 Santai county people’s hospital, Mianyang, 621199 China

**Keywords:** Arthroscopic Bankart repair, Anterior shoulder instability, Recreational athletes, Medium-term outcomes, Magnetic resonance imaging

## Abstract

**Background:**

Extensive research has been conducted to investigate the short-term and long-term outcomes of arthroscopic Bankart repair, yielding varying results across different populations. However, there remains a dearth of studies specifically focused on evaluating outcomes in recreational athletes.

**Methods:**

A retrospective case series study was conducted on recreational athletes who underwent isolated arthroscopic Bankart repair between 2013 and 2021. The primary outcome assessed was recurrent instability, defined as dislocation or subluxation. Secondary outcomes included patient satisfaction, rates of returning to the same sports (RTS) and RTS at preinjury level, and patient-reported outcomes. Evaluation of the Rowe score, Constant score, American Shoulder and Elbow Surgeons score, and VAS pain score were performed. Prognostic factors for recurrent instability, including demographic and clinical characteristics, as well as postoperative magnetic resonance imaging (MRI) appearance of the labrum were analyzed.

**Results:**

A total of 191 patients met the selection criteria, with 150 (78.5%) available for the final follow-up. Recurrent instability occurred in 10.7% of patients, with a mean follow-up duration of 4.1 years. Younger age at surgery and more critical glenoid bone loss were significantly associated with recurrent instability (*p* = .038 and *p* = .011, respectively). The satisfaction rate regarding surgery was 90.0%. Rates of return to the same sports (RTS) and RTS at preinjury level were 82.0% and 49.3%, respectively. Clinical outcomes measured at the final follow-up were as follows: Rowe score − 92.8; Constant score − 98.0; ASES score − 98.3; VAS pain score − 0.2. Patients with recurrent instability had significantly inferior outcomes in terms of satisfaction rate, RTS at preinjury level rate, Rowe score, and Constant score (*p* = .000, *p* = .039, *p* = .000, and *p* = .015, respectively). A total of thirty-seven patients underwent MRI examination six months after surgery in our institution. The T2-weighted anterior labrum morphology was found to be poorer in patients with recurrent instability. No significant difference was observed between patients with or without recurrent instability in terms of anterior Slope, anterior labral glenoid height index (LGHI), inferior Slope, inferior LGHI, and T2-weighted inferior labrum morphology.

**Conclusion:**

Arthroscopic Bankart repair can yield satisfactory medium-term outcomes for recreational athletes. Younger age at surgery, more critical glenoid bone loss, and poorer T2-weighted anterior labrum morphology assessed six months postoperatively were significantly associated with recurrent instability.

**Supplementary Information:**

The online version contains supplementary material available at 10.1186/s13102-024-00925-2.

## Introduction

Anterior shoulder instability (ASI) is a prevalent injury that can significantly impact daily life and sports performance, with reported incidence rates ranging from 0.08 to 0.2 per 1000 person-years in the general population [1, 2]. Surgical intervention is generally recommended following the initial dislocation due to the suboptimal success rate associated with conservative treatment approaches [3, 4].

Currently, arthroscopic Bankart repair (ABR) is the most commonly performed surgical stabilization procedure for ASI worldwide [5, 6]. Previous studies evaluating the clinical outcomes of ABR have mainly focused on high-demand patient populations, such as competitive athletes and soldiers, or encompassed highly heterogeneous patient populations [6–8].

There is an abundance of data available regarding the outcomes of ABR in competitive athletes [9–11]. However, a consensus has yet to be reached on the clinical outcomes of ABR in recreational athletes who have not attained a professional or competitive level. Komnos et al. discovered that ABR may yield favorable to excellent long-term clinical results with an acceptable recurrence rate (11.5%) among recreational athletes and laborers [12]. The study by Komnos et al. revealed that, following an average follow-up period of 12.7 years in a population engaged in recreational sports, ABR exhibited subjective apprehension at a rate of 19% and redislocation at a rate of 19% [13]. Recreational athletes, who do not derive their livelihood from competitive sports and are not employed by for-profit organizations, constitute a substantial proportion of routine clinical cases but have received limited attention [14].There is mounting evidence suggesting that the effectiveness of ABR is closely linked to the specific patient population under consideration. A recent systematic review revealed that contact or collision (CC) athletes exhibited higher rates of recurrence compared to non-collision athletes. Moreover, there was considerable variability in reported recurrence rates following ABR across different types of CC sports, ranging from 3–51% [15]. Consequently, it is advocated that the results after ABR in contact athletes should not be reported globally. Therefore, we contend that the outcome of ABR is significantly influenced by the patient population’s level of physical demand, which varies among high-demand and medium-demand (recreational athletes) or low-demand populations. It becomes imperative to focus on studying a particular population to minimize subject heterogeneity.

The aim of this study was to assess the outcomes following ABR in recreational athletes and to identify clinical and radiographic predictors for recurrent instability. We postulated that ABR could achieve favorable outcomes in terms of shoulder stability, patient satisfaction, return to sports, and patient-reported outcomes.

## Materials and methods

### Study design and subjects

This single-institution retrospective case series study was approved by the Ethics Committee on Biomedical Research of our hospital, and informed consent was obtained from all patients.

We conducted a follow-up on patients with recurrent anterior shoulder instability who underwent ABR in our department between 2013 and 2022. Patients were included if they met the following inclusion criteria: (1) recreational athletes participating regularly in sports at a non-elite or non-professional level [14]; (2) diagnosed with recurrent anterior shoulder instability; (3) underwent isolated ABR; (4) followed up for more than 1 year. Exclusion criteria included: (1) posterior or multidirectional shoulder instability; (2) previous surgery on the same shoulder; (3) concomitant rotator cuff tear or frozen shoulder; (4) glenoid bone loss > 25% as determined by computed tomography; (5) neurological disorders affecting the shoulder joint; and (6) unavailable for the final follow-up due to various reasons.

**Fig. 1:**
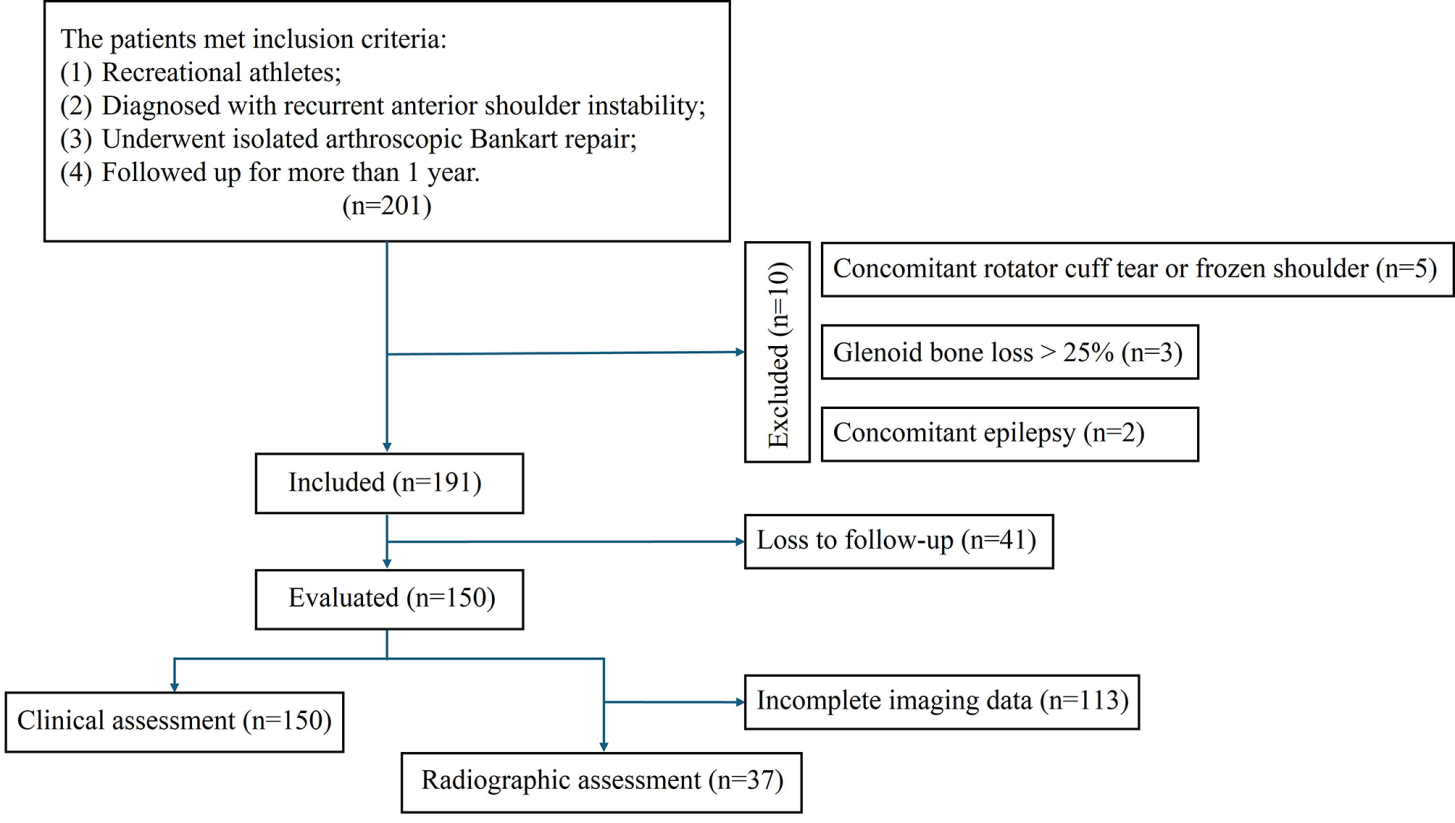
STROBE (Strengthening the Reporting of Observational Studies in Epidemiology) flowchart

**Fig. 2:**
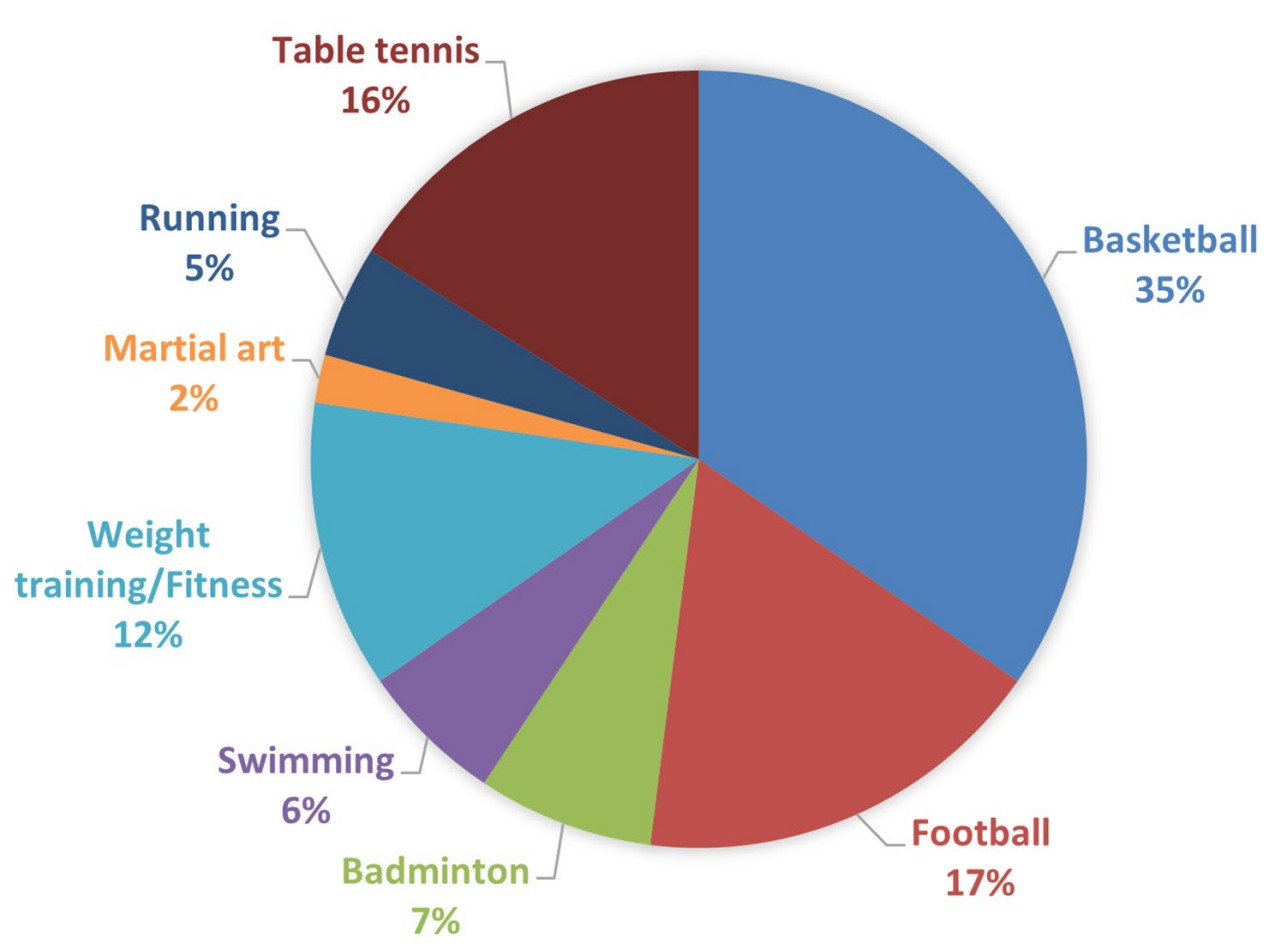
The recreational physical activities practiced by the patients

A total of 191 patients met the selection criteria, with 150 (78.5%) available for the final follow-up assessment (Fig. 1). The baseline characteristics of patients who were followed up completely did not differ significantly from those who were lost to follow-up (Supplementary Table 1). The mean duration of follow-up was 49.0 ± 22.8 months. The average age at primary dislocation was 23.3 ± 7.2 years, and the mean age at surgery was 27.9 ± 8.4 years. No severe complications, such as postoperative hematomas, infections, or neurological damage, were observed during the final follow-up evaluation period. Figure 2 presented the recreational physical activities practiced by the patients. Table 1 provided a summary of patient demographic characteristics and preoperative findings.

**Table 1:** The patients’ demographic and clinical characteristics

Characteristics	Overall (*N* = 150)	RG (*n* = 16)	NG (*n* = 134)	*p* Value
Follow-up, mo	49.0 ± 22.8	49.2 ± 19.3	49.0 ± 23.3	0.978
Male, n (%)	127 (84.7)	16 (100)	111 (82.8)	0.152
Dominant shoulder affected, n (%)	98 (65.3)	10 (62.5)	88 (65.7)	0.801
Age at surgery, y	27.9 ± 8.4	25.1 ± 4.8	28.2 ± 8.7	**0.038**
Age at primary dislocation, y	23.3 ± 7.2	21.6 ± 4.7	23.5 ± 7.5	0.310
Time to surgery, mo	29.5 (12.0, 72.0)	26.5 (18.0, 48.0)	30.0 (12.0, 72.0)	0.920
Number of preoperative dislocations	6 (4, 10)	5 (4, 7.5)	7 (4, 10)	0.303
Number of anchors used	3.8 ± 0.5	3.9 ± 0.3	3.8 ± 0.5	0.655
Presence of Hill-Saches lesion, n (%)	143 (95.3)	16 (100)	127 (94.8)	1.000
Presence of glenoid bone loss, n (%)	126 (84.0)	16 (100)	110 (82.1)	0.137
Proportion of glenoid bone loss, %	9.0 ± 5.5	12.3 ± 3.5	8.6 ± 5.6	**0.011**

RG: recurrent instability group; NG: no recurrent instability group; y: years; mo: months;

### Evaluation

#### Clinical assessment

The patients’ demographic data, including age, gender, and affected dominant shoulder, were routinely collected upon admission. Additionally, medical history information such as age at first dislocation and number of dislocations was recorded. Preoperative scores for Rowe score [16, 17], Constant score [18, 19], American Shoulder and Elbow Surgeons (ASES) score [16, 20], and visual analog scale (VAS) for subjective pain were obtained during this period. Surgical data was extracted from medical records. Clinical assessments at the final follow-up were conducted by an independent investigator through telephonic interviews [13, 21]. Patients were queried regarding shoulder stability status, shoulder function and pain levels, as well as their ability to return to sports activities. Patients provided a qualitative subjective evaluation of surgical satisfaction based on the aforementioned results - either satisfied or dissatisfied. Recurrent instability was defined as postoperative shoulder dislocation or subluxation events. Postoperative functional scores, including the Rowe score, Constant score, and ASES score, were completed by patients using standardized questionnaires along with a 10-point VAS for subjective pain assessment.

#### Radiographic assessment

The glenoid bone defect size was routinely assessed upon admission using three-dimensional CT reconstruction, specifically by measuring the sagittal view of the glenoid. The glenoid diameter and width of the defect were determined using the perfect circle technique [22]. The percentage of glenoid bone defect was calculated according to established literature methods, where it is defined as the ratio between the width of the defect and the glenoid diameter [23, 24].

All patients were recommended to undergo an MRI examination six months post-surgery for the assessment of labrum recovery and potential sports resumption. The final follow-up involved retrieving postoperative MRI data from our hospital’s imaging system. Two blinded investigators (radiologist and orthopedic surgeon) performed all MRI measurements. Labral glenoid height index (LGHI) and labral slope were evaluated following the methods described by Yoo et al. [25] LGHI was calculated as the ratio of labral height (LH) to glenoid height (GH), while labral slope was measured as the angle between the tangent at the lowest point of the glenoid cavity and the tip of the maximum labral height. LH and GH were defined as the maximum distance to the lowest portion of the glenoid cavity. Labrum interior morphology was assessed and graded based on the literature published by Randelli et al. [26] Measurements were conducted using transaxial PDW EXP-weighted images for anterior capsulolabral complex, and coronal T2-weighted images for inferior area.

### Surgical procedure and postoperative rehabilitation

The surgical procedures were performed exclusively by a senior surgeon (X.T.). Patients underwent the procedure under general anesthesia and assumed a lateral decubitus position with the aid of a traction system. Following standard sterile preparation and draping, the posterior, anterosuperior and anteroinferior portals were established. A thorough exploration of the shoulder joint was conducted to confirm the presence of Bankart lesion as well as any concomitant injuries such as superior labral anterior posterior (SLAP) lesion, chondral damage, or rotator cuff tear. The hyperplastic synovial membrane within the joint cavity was excised while releasing the adhesive capsule-labrum complex along the glenoid edge from 2 to 6 o’clock position on the right side. Subsequently, decortication of bone was performed to prepare an actively bleeding surface on the anterior glenoid rim. Depending on tear characteristics observed during arthroscopy, 3 to 4 single/double-thread suture anchors were inserted from 6 to 2 o’clock position on the right side. Fixation of capsule-labrum complex involved lifting and tightening sutures followed by sequential knotting before trimming off excess suture material.All patients adhered to a standardized rehabilitation protocol, necessitating the placement of the operated arm in a sling at 30° abduction for a duration of 4 to 6 weeks post-surgery. During this initial phase, patients were allowed controlled flexion and extension movements of the elbow and wrist. Subsequently, gentle passive range of motion exercises were started until normal shoulder joint movement was restored, performed twice daily in the morning and evening. During each training session, the shoulder joint was mobilized to its maximum tolerable pain threshold in all directions and held for 15 s. However, extreme abduction and external rotation are strictly prohibited within the first three months after surgery. Strengthening exercises were started 12 weeks after the surgery using graduated elastic bands. Follow-up visits occurred at postoperative intervals of 2 and 6 weeks, as well as at 3, 6, and 12 months. Individualized rehabilitation guidance was provided during these visits with consideration given to each patient’s unique recovery progress. The decision to resume sports activities was based on each patient’s functional recovery.

### Statistical analysis

The statistical analysis was conducted using SPSS software version 22.0 (SPSS Inc., Chicago, IL, USA). Normal distribution of data was tested using the Kolmogorov-Smirnov test. Continuous variables were presented as means ± standard deviations or medians (interquartile ranges), while categorical variables were reported as frequencies. The independent sample t test was employed for comparing normally distributed data, whereas the Mann-Whitney U test was utilized for analyzing non-normally distributed data. For dichotomous data, the chi-square and Fisher’s exact tests were applied. Univariate analysis was performed to elucidate the risk factors associated with recurrent instability. A significance level of *P* < .05 denoted statistical significance.

## Results

### Recurrent instability

A total of 16 patients (10.7%) experienced recurrent instability, comprising 8 redislocations and 8 subluxations; none of these cases underwent revision surgery. The first redislocation occurred at an average of 35.3 ± 13.6 months (range: 16–55). Among the eight cases of redislocation, four were attributed to collision sports, two to overhead sports, and two to falls.

The univariate analysis revealed that younger age at surgery and more critical glenoid bone loss were identified as significant risk factors for recurrent instability (*p* = .038; *p* = .011). However, gender, dominant shoulder affected, age at primary dislocation, time to surgery, number of preoperative dislocations and anchors used, or presence of Hill-Sachs lesion and glenoid bone loss did not show any association with postoperative recurrent instability (Table 1).

### Subjective patient satisfaction

The final follow-up revealed a subjective satisfaction rate of 90.0% among patients. Notably, patients with recurrent instability exhibited significantly lower levels of satisfaction compared to those without recurrent instability (*p* = .000) (Table 2).

### Return to sports

A total of 123 patients (82.0%) reported successful return to the same sports (RTS) following surgery, while 74 patients (49.3%) achieved RTS at their preinjury level. However, the rate of achieving RTS at preinjury level was significantly lower in patients with recurrent instability compared to those without recurrent instability (*p* = .039) (Table 2).

### Patient-reported outcomes

At the final follow-up, the patient-reported outcomes, including Rowe score, Constant score, AESE score and VAS for pain, demonstrated favorable results. All four scores exhibited significant improvement in patients without recurrent instability; however, the Rowe score did not show improvement in patients with recurrent instability. Patients with recurrent instability had significantly lower postoperative Rowe and Constant scores compared to those without recurrent instability (*p* = .000; *p* = .015) (Table 2).

**Table 2:** Clinical outcomes at the final follow-up assessment

Results	Total (*n* = 150)	RG (*n* = 16)			NG (*n* = 134)			*p* value
Recurrent instability, n (%)	16 (10.7)	16 (100)			-			-
Redislocation, n (%)	8 (5.3)	8 (50)			-			-
Subluxation, n (%)	8 (5.3)	8 (50)			-			-
Satisfied, n (%)	135 (90.0)	5 (31.3)			130 (97.0)			**0.000**
RTS, n (%)	123 (82.0)	10 (62.5)			113 (84.3)			0.071
RTS at preinjury level, n (%)	74 (49.3)	4 (25.0)			70 (52.2)			**0.039**
		Preoperative	Postoperative	p	Preoperative	Postoperative	p	
Rowe score	92.8 ± 15.8	45.9 ± 6.1	49.7 ± 10.2	0.237	45.5 ± 8.4	98.0 ± 4.2	0.000	**0.000**
Constant score	98.0 ± 3.8	73.4 ± 15.5	93.1 ± 8.0	0.001	84.3 ± 13.8	98.6 ± 2.4	0.000	**0.015**
AESE score	98.3 ± 3.2	77.2 ± 8.8	95.8 ± 6.0	0.000	79.0 ± 8.4	98.7 ± 2.5	0.000	0.075
VAS score	0.2 ± 0.5	1.9 ± 1.3	0.3 ± 0.7	0.001	1.3 ± 1.0	0.2 ± 0.4	0.000	0.494

RG: recurrent instability group; NG: no recurrent instability group; RTS: return to the same sports

### Postoperative MRI assessment

A total of 37 patients underwent MRI examination at our institution 6.0 ± 1.3 months after surgery. Notably, the group that received MRI had a significantly shorter follow-up duration compared to those who did not undergo this procedure (Supplementary Table 2). Postoperative MRI assessments revealed similar data, including anterior labral slope (aSlope), anterior LGIH (aLGHI), inferior labral slope (iSlope), inferior LGHI (iLGHI) and T2-weighted inferior labrum morphology, in patients with and without recurrent instability. However, a significant difference was observed in the T2-weighted anterior labrum morphology between the two groups (*p* = .029). The status of the anterior labrum was significantly poorer in patients with recurrent instability (Table 3). All suture anchors for Bankart repair were detected intact at their original drill holes without any instances of total or partial dislocations.

**Table 3:** The postoperative MRI assessment of the anterior and inferior labrum

	Overall (*N* = 37)	RG (*n* = 6)	NG (*n* = 31)	*p* Value
Interval between surgery and assessment, mo	6.0 ± 1.3	6.5 ± 1.0	5.9 ± 1.3	0.378
aSlope, deg	25.2 ± 2.8	23.4 ± 3.9	25.5 ± 2.5	0.092
aLGHI	2.6 ± 0.2	2.5 ± 0.2	2.7 ± 0.3	0.226
T2-weighted anterior labrum morphology				**0.020**
Grade 0	7	0	7	
Grade I	19	2	17	
Grade II	10	3	7	
Grade III	1	1	0	
iSlope, deg	26.8 ± 2.9	26.7 ± 2.9	26.9 ± 3.0	0.899
iLGHI	2.7 ± 0.4	2.5 ± 0.3	2.7 ± 0.4	0.278
T2-weighted inferior labrum morphology				0.137
Grade 0	5	0	5	
Grade I	26	4	22	
Grade II	6	2	4	
Grade III	0	0	0	

RG: recurrent instability group; NG: no recurrent instability group; aSlope: anterior labral slope; aLGHI: anterior labral glenoid height index; iSlope: inferior labral slope; iLGHI: inferior labral glenoid height index; Grade 0: homogenous structure with normal morphology; Grade I: punctiform or intralabral nodular hypersignal with normal morphology; Grade II: linear hypersignal extended to labrum surface with changed morphology; Grade III: complex hypersignal multiply extended to labrum surface with disrupted morphology

## Discussion

The primary finding of this study demonstrated favorable medium-term outcomes in recreational athletes who underwent ABR, with a mean follow-up duration of 4.1 years. Only 5.3% of patients experienced redislocation, resulting in an overall satisfaction rate of 90.0%. The rates of return to sport (RTS) and RTS at preinjury level were found to be 82.0% and 49.3%, respectively. Notably, younger age at the time of surgery, greater glenoid bone loss, and poorer T2-weighted anterior labrum morphology assessed six months postoperatively were significantly associated with recurrent instability.

The primary objective of the surgical stabilization procedure is to restore shoulder stability and prevent recurrent dislocation. A study conducted by Saper et al., with an average follow-up period of 6.3 years, demonstrated a redislocation rate of 10.3% among 37 adolescent athletes [27]. Calvisi et al., in their research involving 22 professional rugby players with a mean follow-up duration of 3.4 years, reported a redislocation rate of 13.6% [7]. However, in a long-term study encompassing the general population over a follow-up period of 13 years, the redislocation rate was found to be only 9.6% [28]. These findings suggest that the incidence of redislocation following ABR is influenced by patient demographics; therefore, it is imperative to investigate different patient populations separately to enhance the applicability of conclusions drawn from such studies. In our study, we observed a mere 5.3% redislocation rate among patients with medium-demand lifestyles at an average follow-up duration of 4.1 years, indicating promising long-term outcomes in terms of recurrent instability.

In summary, our study demonstrates that ABR can achieve satisfactory medium-term efficacy in terms of patient satisfaction, patient-reported outcomes (PRO), and return to sports, which is consistent with findings from previously published studies [21, 29, 30]. A recent study by Bauer et al. [6], involving 46 athletes with a mean follow-up of 14 years, reported a high satisfaction rate of 91.3%. In that study, the Constant score and WOSI score showed favorable results, while the Rowe score indicated moderate outcomes with 84.4% of patients returning to sports. Notably, the rate of return to preinjury sport level was only 49.3% in this study, lower than that reported in other studies. This discrepancy can be attributed to two factors: firstly, a lack of professional guidance for safe return to sports among most patients; and secondly, a reduction in sports participation due to fear of redislocation. Pasqualini et al. discovered that patients who were not psychologically prepared for resuming sports activities following shoulder instability surgery experienced worse clinical outcomes in terms of pain and had a higher risk of recurrence [31]. This suggests that incorporating psychological intervention to alleviate fear is crucial for improving surgical outcomes during postoperative rehabilitation [32].

Furthermore, this study has confirmed the negative impact of recurrent instability on clinical outcome at the final follow-up, as previously reported [6]. Patients with recurrent instability exhibited significantly lower rates of satisfaction and return to sport at preinjury level, as well as inferior Rowe scores and Constant scores compared to those without recurrent instability. Therefore, strict adherence to the indications for ABR is crucial in order to prevent recurrence. For high-risk instability patients, more invasive procedures such as the Latarjet procedure should be considered [33].

Identification of risk factors for recurrence is crucial in reducing adverse outcomes following surgical stabilization procedures. Currently recognized risk factors include age ≤ 20 years, participation in competitive sports, and bony lesions [34–36]. In our study, younger age at surgery and more critical glenoid bone loss were significantly associated with recurrent instability. However, other major factors such as the age at primary dislocation, number of preoperative dislocations and presence of Hill-Sachs lesion did not show any association. The average glenoid bone loss among patients with recurrent instability was found to be 12.3% in this study, which closely approximates the subcritical threshold of 13.5% as reported in previous literature [37]. Our findings suggest that patients with subcritical glenoid bone loss may benefit from bony augmentation surgery to restore native anatomy [38, 39].

The protocol for MRI assessment of labrum integrity following ABR has been established in previous studies. Quantitative evaluation of the labrum structure was conducted through measurements of the labral slope and LGHI, while qualitative evaluation was performed using the Randelli classification. Yoo et al. measured labral height and slope on axial and oblique coronal images at the anteroinferior portion of the glenoid [25]. They reported a significant increase between the postoperative week 6 and preoperative period in all four parameters, which maintained at 6 months postoperatively. Bock et al. performed bilateral MRI to assess labrum restoration in long-term [26]. Their results, with a mean follow-up period of 8.8 years, showed comparable parameters for anteroinferior slope and LGHI between the operated and control side, but significantly poorer T2-weighted anterior labrum morphology on the operated side. In contrast to previous studies, our findings revealed no significant difference in anteroinferior labral slope and LGHI parameters at postoperative 6 months between patients with and without recurrent instability. However, we did observe poorer T2-weighted anterior labrum morphology at 6 months postoperatively in patients with recurrent instability. These results suggested that ABR can effectively restore structural integrity of the labrum, however, poor T2-weighted anterior labrum morphology assessed at postoperative 6 months may be associated with recurrent instability. Therefore, it is recommended that these patients temporarily avoid high-risk factors for recurrence such as collision sports or falls.

A recent study conducted by Pasqualini et al. revealed considerable variability in the minimal clinically important difference and patient acceptable symptom state thresholds following ABR, when accounting for various patient characteristics such as sex, age, sports participation, and athlete type [40]. This emphasizes the importance of considering individual patient-specific attributes during the assessment of ABR efficacy. To our knowledge, no systematic review has been conducted on postoperative outcomes of ABR specifically in recreational athletes. This retrospective case series study boasts the largest sample size within this field.

This study had several limitations. Firstly, it was a single-institution case series with a 21.5% loss to follow-up rate, which introduced bias into the results. Secondly, due to the relatively short mean follow-up period of 4.1 years, the assessment of osteoarthritis signs was not conducted in this study, potentially limiting the evaluation of long-term complications following ABR. Thirdly, only a quarter of the patients underwent MRI at our institution 6 months postoperatively; although this finding reflects positive outcomes following ABR, missing data may have influenced the results of postoperative MRI assessment. Fourthly, most of the patients were followed up through telephone surveys, precluding the possibility of conducting a precise physical examination on them.

## Conclusion

Arthroscopic Bankart repair can achieve satisfactory medium-term outcomes for recreational athletes. The younger age at surgery, more critical glenoid bone loss, and poorer T2-weighted anterior labrum morphology assessed at 6 months postoperatively were significantly associated with recurrent instability.

### Electronic supplementary material

Below is the link to the electronic supplementary material.

Supplementary Material 1

Supplementary Material 2

## Data Availability

The datasets used and analyzed during the current study are available from the corresponding author on reasonable request.

## Abbreviations

MRI: Magnetic resonance imaging
RTS: Return to the same sports
LGHI: Labral glenoid height index
ASI: Anterior shoulder instability
ASES: American Shoulder and Elbow Surgeons
VAS: Visual analog scale
LH: Labral height
GH: Glenoid height
SLAP: Superior labral anterior posterior
aSlope: Anterior labral slope
iSlope: Inferior labral slope
PRO: Patient-reported outcomes
