# Supplementary material for: Prognostic factors for recurrent instability in recreational athletes following arthroscopic Bankart repair: a retrospective study with an average 4.1-year follow-up
Source: BMC Sports Sci Med Rehabil. 2024 Jun 24;16:140. doi: 10.1186/s13102-024-00925-2 (PMC11194893; doi:10.1186/s13102-024-00925-2)
Supplement: Supplementary file 1 — Supplementary Material 1 [file 13102_2024_925_MOESM1_ESM.docx]

Supplementary table 1 The baseline characteristics of patients with complete follow-up and those lost to follow-up

| Characteristics | Complete follow-up (N = 150) | Lost to follow-up (n = 41) | p Value |
| --- | --- | --- | --- |
| Male, n (%) | 127 (84.7) | 36 (87.8) | .615 |
| Dominant shoulder affected, n (%) | 98 (65.3) | 31 (75.6) | .213 |
| Age at surgery, y | 27.9 ± 8.4 | 27.7 ± 11.5 | .921 |
| Age at primary dislocation, y | 23.3 ± 7.2 | 23.4 ± 8.4 | .960 |
| Time to surgery, mo | 29.5 (12.0, 72.0) | 24.0 (6.5, 60.0) | .375 |
| Number of preoperative dislocations | 6 (4, 10) | 8 (3.5, 15) | .498 |
| Number of anchors used | 3.8 ± 0.5 | 3.9 ± 0.5 | .244 |
| Presence of Hill-Saches lesion, n (%) | 143 (95.3) | 38 (92.7) | .780 |
| Presence of glenoid bone loss, n (%) | 126 (84.0) | 30 (73.2) | .112 |
| Proportion of glenoid bone loss, % | 9.0 ± 5.5 | 8.5 ± 7.0 | .606 |

y: years; mo: months;
