# Supplementary material for: Prognostic factors for recurrent instability in recreational athletes following arthroscopic Bankart repair: a retrospective study with an average 4.1-year follow-up
Source: BMC Sports Sci Med Rehabil. 2024 Jun 24;16:140. doi: 10.1186/s13102-024-00925-2 (PMC11194893; doi:10.1186/s13102-024-00925-2)
Supplement: Supplementary file 2 — Supplementary Material 2 [file 13102_2024_925_MOESM2_ESM.docx]

Supplementary table 2 The baseline characteristics of patients who underwent MRI and those who did not

| Characteristics | Complete MRI assessment (N = 37) | Incomplete MRI assessment (n = 113) | p Value |
| --- | --- | --- | --- |
| Follow-up, mo | 43.4 ± 17.4 | 50.9 ± 24.1 | **.042** |
| Male, n (%) | 30 (81.1) | 97 (85.8) | .486 |
| Dominant shoulder affected, n (%) | 24 (64.9) | 74 (65.5) | .945 |
| Age at surgery, y | 27.5 ± 8.3 | 28.0 ± 8.5 | .748 |
| Age at primary dislocation, y | 22.1 ± 6.4 | 23.7 ± 7.4 | .228 |
| Time to surgery, mo | 30.0 (12.0, 108.0) | 29.0 (12.0, 72.0) | .619 |
| Number of preoperative dislocations | 6.0 (3.5, 9.5) | 6.0 (4.0, 11.0) | .726 |
| Number of anchors used | 3.9 ± 0.5 | 3.8 ± 0.5 | .333 |
| Presence of Hill-Saches lesion, n (%) | 36 (97.3) | 107 (94.7) | .839 |
| Presence of glenoid bone loss, n (%) | 33 (89.2) | 93 (82.3) | .321 |
| Proportion of glenoid bone loss, % | 9.0 ± 4.3 | 9.0 ± 5.9 | .993 |

mo: months; y: years
